# Association between Adipose Tissue Interleukin-33 and Immunometabolic Markers in Individuals with Varying Degrees of Glycemia

**DOI:** 10.1155/2019/7901062

**Published:** 2019-04-03

**Authors:** Amal Hasan, Shihab Kochumon, Ebaa Al-Ozairi, Jaakko Tuomilehto, Rasheed Ahmad

**Affiliations:** ^1^Research Division, Dasman Diabetes Institute, Dasman 15462, Kuwait; ^2^Medical Division, Dasman Diabetes Institute, Dasman 15462, Kuwait; ^3^Department of Medicine, Faculty of Medicine, Kuwait

## Abstract

**Introduction:**

Interleukin-33 (IL-33), the ligand for the receptor ST2, is abundant in adipose tissue, including preadipocytes, adipocytes, and endothelial cells. The IL-33/ST2 axis is protective against obesity, insulin resistance, and type 2 diabetes (T2D) in animal models. We determined whether adipose tissue IL-33 was associated with glycated hemoglobin (HbA1c), as well as mediators of inflammation and immune regulation and beiging of adipose tissue, among individuals with varying degrees of glycemia.

**Materials and Methods:**

A total of 91 adults with normoglycemia, prediabetes, and T2D were included. After measuring their anthropometric and biochemical parameters, subcutaneous adipose tissue samples were isolated and mRNA expression of cytokines, chemokines, chemokine receptors, pattern recognition receptors, and mediators involved in beiging of adipose tissue were measured.

**Results:**

Adipose tissue IL-33 was inversely associated with HbA1c in individuals with normoglycemia and T2D but not in those with prediabetes and was inversely correlated with fasting plasma glucose in individuals with T2D and with a better glycemic control. IL-33-to-ST2 ratio was inversely correlated with HbA1c in individuals with normoglycemia but not in those with prediabetes or T2D. IL-33 was directly associated with ST2, CD302, fibrinogen-like protein 2 (FGL2), and PR domain containing 16 (PRDM16) but inversely correlated with chemokine (C-C motif) ligand (CCL) 7 and CCL8 in individuals with normoglycemia. Similarly, IL-33 was directly associated with ST2, CD302, FGL2, PRDM16, and, additionally, toll-like receptor (TLR) 3 and IL-12A in individuals with T2D. However, IL-33 was not associated with any of these mediators but was directly and strongly associated with TLR9 in individuals with prediabetes.

**Conclusions:**

IL-33 and/or IL-33/ST2 dynamics and biological functions may play a role in overall glycemia among humans and may represent a novel target by which glucose-lowering managements confer their beneficial effects.

## 1. Introduction

A state of chronic low-grade inflammation in the adipose tissue plays a major role in the development of metabolic disease, such as insulin resistance and type 2 diabetes (T2D). Although adiposity is often associated with metainflammation and metabolic disorders [1, 2], a group of obese individuals remains metabolically healthy [3]; this reflects the complex and multifactorial nature of metabolic disease development. Adipose tissue is a key regulator of energy balance and is generally divided into two broad categories, namely, white adipose tissue and brown adipose tissue [4–6]. In addition, white adipose tissue can convert to a brown-like depot, termed “beige” adipose [7, 8]. Brown and beige adipose tissues dissipate energy and influence glucose and VLDL-TG metabolism [9, 10], which may protect against metabolic diseases, such as T2D. Immune cells and their mediators have emerged as a major axis of metabolic regulation [11–13], which has given rise to a new paradigm in immune regulation of metabolism. Type 1 immune responses are involved in the induction/exacerbation of metabolic dysfunction, such as obesity and diabetes, whereas type 2 immune programs are responsible for maintaining and fine-tuning tissue function [13–15].

Interleukin-33 (IL-33) is an IL-1-like cytokine that signals via the IL-1 receptor-related protein ST2 and plays an important role in Th2-associated immune responses. IL-33, in addition to being secreted as a cytokine, is constitutively expressed in the nucleus of various cell types including preadipocytes and adipocytes, endothelial cells, epithelial cells [16–18], and fibroblast-like reticular cells [19–23] and may also have transcriptional regulatory capacity [16]. ST2, together with its coreceptor IL-1RAcP [24], form the IL-33 receptor complex. Two splice variants of the ST2 gene have been described; one encodes for a transmembrane isoform (ST2L), and the other one encodes for a secreted soluble isoform (sST2) [25]. ST2L confers the biological effects of IL-33, whereas sST2 serves as an antagonistic decoy receptor [26]. ST2L, initially regarded as a selective marker for Th2 cells [25, 27–30], is also expressed by various other immune cells, including Treg cells [31, 32], Th9 cells [33, 34], mast cells [28, 35–37], basophiles, eosinophils [38, 39], monocytes [40], macrophages [41, 42], innate lymphoid cell type 2 (ILC2) [43, 44], dendritic cells [45, 46], neutrophils [38, 47], B1 B cells [48], invariant natural killer T (iNKT) cells, NK cells [37, 49], and Tc1 T cells [29, 50, 51].

The IL-33/ST2 pathway plays a protective role against obesity, insulin resistance, and T2D in animal models [18, 52, 53]. The mechanisms by which IL-33 exerts these protective metabolic effects include the reduction of resistin expression [18], accumulation of protective Th2 cells and associated cytokines, and polarization of resident macrophages toward a protective alternatively activated phenotype [18, 52]. These findings have been supported by *in vitro* studies showing that treatment with IL-33 induces the production of protective Th2 cytokines (mainly IL-5 and IL-13), reduces lipid storage, and decreases the expression of genes associated with lipid metabolism and adipogenesis [18, 52]. Moreover, IL-33 administration to diabetic obese (*ob*/*ob*) mice results in the reduction of adiposity and fasting glucose, as well as improved glucose tolerance and insulin levels [18, 52], whereas feeding ST2^−/−^ mice a high-fat diet leads to increased body weight and fat mass, as well as impaired insulin secretion and glucose regulation [18, 52, 53]. In addition, recent evidence suggests that circulating IL-33 may confer protective metabolic effects (body mass index (BMI), lipid profile, and glycated hemoglobin (HbA1c)) in humans, specifically, in individuals with normal weight and glycemia but not in those with obesity and/or T2D [54]. However, little is known about the metabolic effects of adipose tissue IL-33 in humans. Therefore, we investigated the expression level of IL-33 (and ST2) in the adipose tissue, as well as its association with overall glycemia (measured by HbA1c), mediators of inflammation and immune regulation, and beiging of adipose tissue, among individuals with normoglycemia, prediabetes, and T2D.

## 2. Materials and Methods

### 2.1. Study Participants

The study was conducted in accordance with the ethical principles of the Declaration of Helsinki and approved by the Ethical Review Committee of Dasman Diabetes Institute, State of Kuwait. All participants had provided written informed consent. The study included 91 adults between the ages of 23 and 72, which were classified based on their HbA1c (in accordance with the American Diabetes Association criteria [55]) into three groups, namely, normoglycemia (HbA1c < 5.7%), prediabetes (HbA1c 5.7-6.4%; not on glucose-lowering medications), and T2D (a diagnosis of T2D with an HbA1c ≥ 6.5%; on any type of glucose-lowering medications) [55–57]. Comorbid health conditions included hypertension, dyslipidemia, cardiovascular disease, kidney disease, and neuropathy.

### 2.2. Anthropometric and Biochemical Measurements

After measuring participant's body weight (kg) and height (to the nearest 0.5 cm), BMI was then calculated as weight/height^2^ (kg/m^2^) and used as an overall index of adiposity. The waist-to-hip ratios were calculated, and the whole-body composition including percentage of body fat (PBF), soft lean mass (SLM), and total body water (TBW) was measured using the IOI 353 Body Composition Analyzer (Jawon Medical). Fasting blood samples were obtained, and plasma glucose, serum total cholesterol, triglycerides (TG), and high-density lipoprotein (HDL) cholesterol were measured using the Siemens Dimension RXL chemistry analyzer (Diamond Diagnostics, Holliston, MA, USA). Low-density lipoprotein (LDL) cholesterol was also estimated. Glycated hemoglobin (HbA1c) was determined using Variant™ (Bio-Rad, Hercules, CA, USA). High-sensitivity C-reactive protein (hsCRP) was measured using the ELISA kit (BioVendor, USA). Optimal values for TG (<1.7 mmol/L), total cholesterol (<5.2 mmol/L), LDL cholesterol (<3.3 mmol/L), and HDL cholesterol (>1.03 mmol/L) were based on the American Heart Association's guidelines. Anthropometric and biochemical parameters are shown in Table 1.

### 2.3. Collection of Subcutaneous Adipose Tissue Samples

Human adipose tissues (~0.5 g) were collected via a standard surgical procedure from abdominal subcutaneous fat pads situated lateral to the umbilicus. Briefly, the periumbilical area was sterilized using alcohol swabs and then locally anesthetized using 2% lidocaine (2 mL). A small superficial skin incision (0.5 cm) was then made; after which, fat tissue was collected using punch biopsy. Freshly collected adipose tissues (~50–100 mg) were preserved in RNAlater or embedded in optimal cutting temperature (OCT) compound and stored at −80°C until use.

### 2.4. RNA Isolation from Adipose Tissue Samples

Total cellular RNA was purified using the RNeasy kit (Qiagen, Valencia, CA, USA) as per manufacturer's instructions. Briefly, adipose tissue samples (preserved in RNAlater or embedded in OCT) were thawed and homogenized in Qiazol lysis solution (Qiagen, Valencia, CA, USA) using TissueRuptor (Qiagen, Hilden, Germany) at 33,000 rpm for 40 s. The homogenates were then treated with chloroform and separated into aqueous and organic phases by centrifugation at 12,000 ×g for 15 min at 4°C. After the upper aqueous RNA phase was collected, 70% ethanol was then added. Thereafter, the sample was applied to an RNeasy spin column to allow total RNA binding with the membrane and to wash out phenol and other contaminants. High-quality RNA was then eluted in RNase-free water. The quantity and quality of the isolated RNA were determined using the Epoch™ Spectrophotometer System (BioTek, Winooski, VT, USA) and formaldehyde–agarose gel electrophoresis, respectively.

### 2.5. Real-Time Reverse Transcription-Polymerase Chain Reaction

Real-time reverse transcription-polymerase chain reaction was conducted, as previously described [58]. Briefly, RNA samples (1 *μ*g each) were reverse transcribed to cDNA using random hexamer primers and TaqMan reverse transcription reagents (High-Capacity cDNA Reverse Transcription kit; Applied Biosystems, CA, USA). For real-time reverse transcription-polymerase chain reaction (RT-PCR), cDNA (50 ng) was amplified using TaqMan® Gene Expression MasterMix (Applied Biosystems, CA, USA) and gene-specific 20x TaqMan Gene Expression Assays (Applied Biosystems, CA, USA) containing forward and reverse primers and a target-specific TaqMan® minor groove binder (MGB) probe labeled with 6-fluorescein amidite dye at the 5′ end and a nonfluorescent quencher MGB at the 3′ end of the probe for 40 cycles of PCR reaction using a 7500 Fast Real-Time PCR System (Applied Biosystems, CA, USA). Each cycle consisted of a denaturation phase for 15 s at 95°C and an annealing/extension phase for 1 min at 60°C. The cycle was started after uracil DNA glycosylase activation (50°C for 2 min) and AmpliTaq Gold enzyme activation (95°C for 10 min). Amplified glyceraldehyde 3-phosphate dehydrogenase (GAPDH) expression was used as an internal control to normalize differences between individual samples. The comparative C_T_ method was used to analyze the relative gene expression of various mediators using the formula 2^-ΔΔC^_T_ whereby ΔΔC_T_ = [C_T_ gene of interest–C_T_ endogenous control; ΔC_T_]–normal weight control with highest ΔC_T_. In this regard, the relative mRNA fold expression for each specific gene was calculated by first normalizing the C_T_ values to an endogenous control (GAPDH) (ΔC_T_) and then normalizing the ΔC_T_ of each gene expression to the highest ΔC_T_ of normal weight control. The assay IDs (TaqMan® Assays, Thermo Fisher Scientific) of the analyzed genes are shown in Supplementary Materials (available here).

### 2.6. Statistical Methods

The GraphPad Prism software (version 7.04; San Diego, CA, USA) was used for all statistical analyses. Correlation analysis was conducted using nonparametric Spearman's *r* test, and results were validated using an extra test wherein an estimated cutoff value for IL-33 (median mRNA expression of IL-33 in individuals with normoglycemia = 1.5) was used to compare between low and high IL-33 in terms of the medians of various biomarkers. To compare between two groups of data, the nonparametric Mann–Whitney test was utilized with a *P* value of less than 0.05 being considered statistically significant.

## 3. Results

### 3.1. Adipose Tissue IL-33 and Its Association with ST2

**Figure 1:**
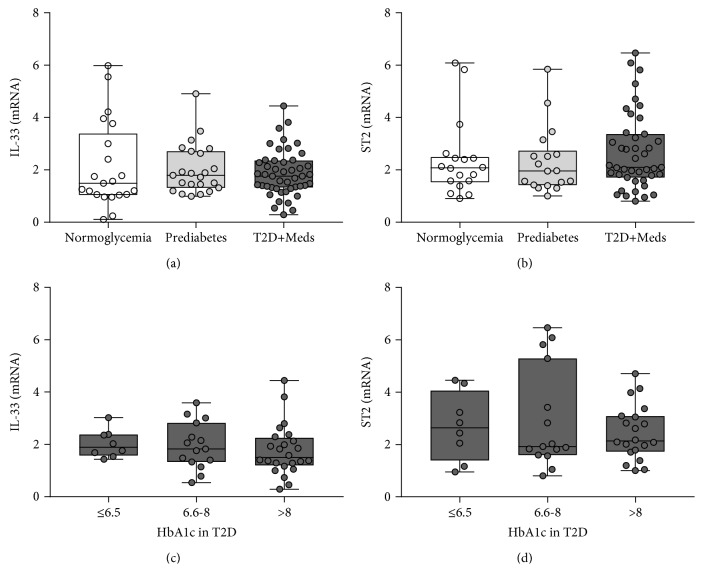
Expression of adipose tissue IL-33 and ST2 among individuals with varying degrees of glycemia. (a) There was no difference in the level of IL-33 among individuals with normoglycemia, prediabetes, or T2D. (b) There was no difference in the level of ST2 among individuals with normoglycemia, prediabetes, or T2D. (c) There was no difference in the level of IL-33 among individuals with T2D and varying degrees of glycemic control. (d) There was no difference in the level of ST2 among individuals with T2D and varying degrees of glycemic control. IL-33: interleukin-33; ST2: suppressor of tumorigenicity 2; HbA1c: glycated hemoglobin; T2D: type 2 diabetes; Meds: glucose-lowering medications.

**Figure 2:**
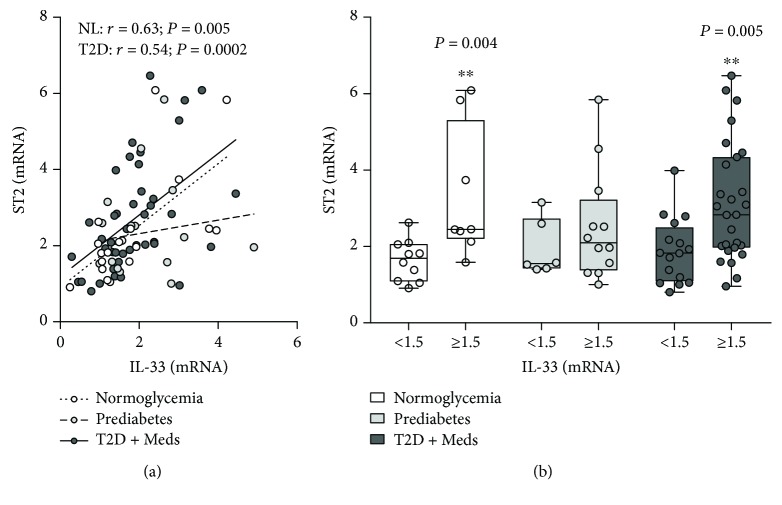
Association between adipose tissue IL-33 and ST2 in individuals with varying degrees of glycemia. (a) IL-33 was directly correlated with ST2 in individuals with normoglycemia (*n* = 18) and T2D (*n* = 43) but not in those with prediabetes (*n* = 17). (b) Higher levels of IL-33 were associated with significantly higher ST2 in individuals with normoglycemia and T2D but not in those with prediabetes. IL-33: interleukin-33; ST2: suppressor of tumorigenicity 2; NL: normoglycemia; T2D: type 2 diabetes; Meds: glucose-lowering medications.

There was no difference in the expression levels of IL-33 or ST2 among individuals with normoglycemia, prediabetes, or T2D (Figures 1(a) and 1(b)). In addition, no difference was observed in the expression levels of IL-33 or ST2 among individuals with T2D who had varying degrees of glycemic control (HbA1c ≤ 6.5, HbA1c 6.6–8.0, HbA1c > 8.0) (Figures 1(c) and 1(d)). IL-33 was directly correlated with ST2 in individuals with normoglycemia (*r* = 0.63; *P* = 0.005; *n* = 18) and T2D (*r* = 0.54; *P* = 0.0002; *n* = 43) but not in those with prediabetes. Similarly, higher levels of IL-33 were associated with significantly higher ST2 in individuals with normoglycemia (*P* = 0.004; median 2.45, *n* = 8 vs. median 1.69, *n* = 10) and T2D (*P* = 0.005; median 2.83, *n* = 27 vs. median 1.83, *n* = 16) but not in those with prediabetes (Figures 2(a) and 2(b)).

### 3.2. Association between Adipose Tissue IL-33 and Clinicometabolic Parameters

**Figure 3:**
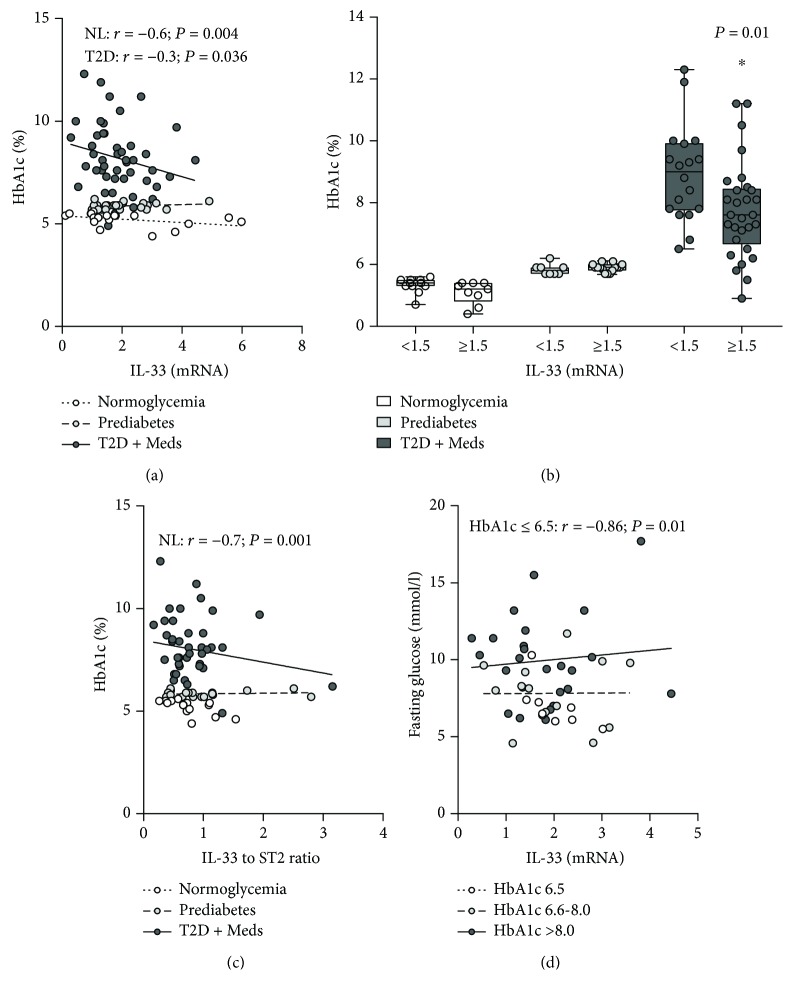
Association between adipose tissue IL-33 and HbA1c (and fasting glucose in T2D) in individuals with varying degrees of glycemia. (a) IL-33 was inversely correlated with HbA1c in individuals with normoglycemia (*n* = 20) and T2D (*n* = 47) but not in those with prediabetes (*n* = 23). (b) Higher levels of IL-33 were associated with lower HbA1c in individuals with normoglycemia (although statistically nonsignificant) and T2D (statistically significant) but not in those with prediabetes. (c) The ratio of IL-33 to ST2 was inversely correlated with HbA1c in individuals with normoglycemia (*n* = 17) but not in those with prediabetes (*n* = 16) or T2D (*n* = 43). (d) IL-33 was inversely correlated with fasting glucose in individuals with T2D who had a better glycemic control (*n* = 8). IL-33: interleukin-33; ST2: suppressor of tumorigenicity 2; HbA1c: glycated hemoglobin; NL: normoglycemia; T2D: type 2 diabetes; Meds: glucose-lowering medications.

IL-33 was inversely correlated with HbA1c in individuals with normoglycemia (*r* = −0.6; *P* = 0.004; *n* = 20) and T2D (*r* = −0.3; *P* = 0.036; *n* = 47) but not in those with prediabetes. Among individuals with normoglycemia, those who had higher levels of IL-33 had lower (although statistically nonsignificant) HbA1c compared with those who had lower levels of IL-33 (*P* = 0.07; median 5.2, *n* = 9 vs. median 5.4, *n* = 11). This pattern was not observed among individuals with prediabetes. Among individuals with T2D, those who had higher levels of IL-33 had significantly lower HbA1c compared with those who had lower levels of IL-33 (*P* = 0.01; median 7.6, *n* = 29 vs. median 9, *n* = 18) (Figures 3(a) and 3(b)). No correlation was observed between ST2 and HbA1c among individuals with normoglycemia, prediabetes, or T2D. The ratio of IL-33 to ST2 was inversely correlated with HbA1c in individuals with normoglycemia (*r* = −0.7; *P* = 0.001; *n* = 17) but not in those with prediabetes or T2D (Figure 3(c)). To determine whether the inverse correlation between IL-33 and HbA1c in people with T2D was confined to individuals with a better glycemic control, they were divided into three groups based on their level of glycemic control (i.e., HbA1c ≤ 6.5, HbA1c 6.6–8.0, and HbA1c > 8.0) and analyzed separately. Accordingly, IL-33 was not correlated with HbA1c in individuals with T2D when stratified for their level of glycemic control. However, IL-33 was inversely correlated with fasting glucose (*r* = −0.86; *P* = 0.01) in individuals with T2D who had a better glycemic control (HbA1c ≤ 6.5; *n* = 8) (Figure 3(d)). No correlation was found between adipose tissue IL-33 and circulating lipids (total cholesterol, LDL, HDL, and TG), BMI, body composition (PBF, SLM %, and TBW %), or waist-to-hip ratio among individuals with normoglycemia, prediabetes, or T2D. No difference in these parameters was observed among individuals with higher versus lower levels of IL-33. Interestingly, IL-33 in individuals with T2D increased with age (*r* = 0.45; *P* = 0.001; *n* = 47; data not shown).

### 3.3. Association between Adipose Tissue IL-33 and Macrophage Markers

**Figure 4:**
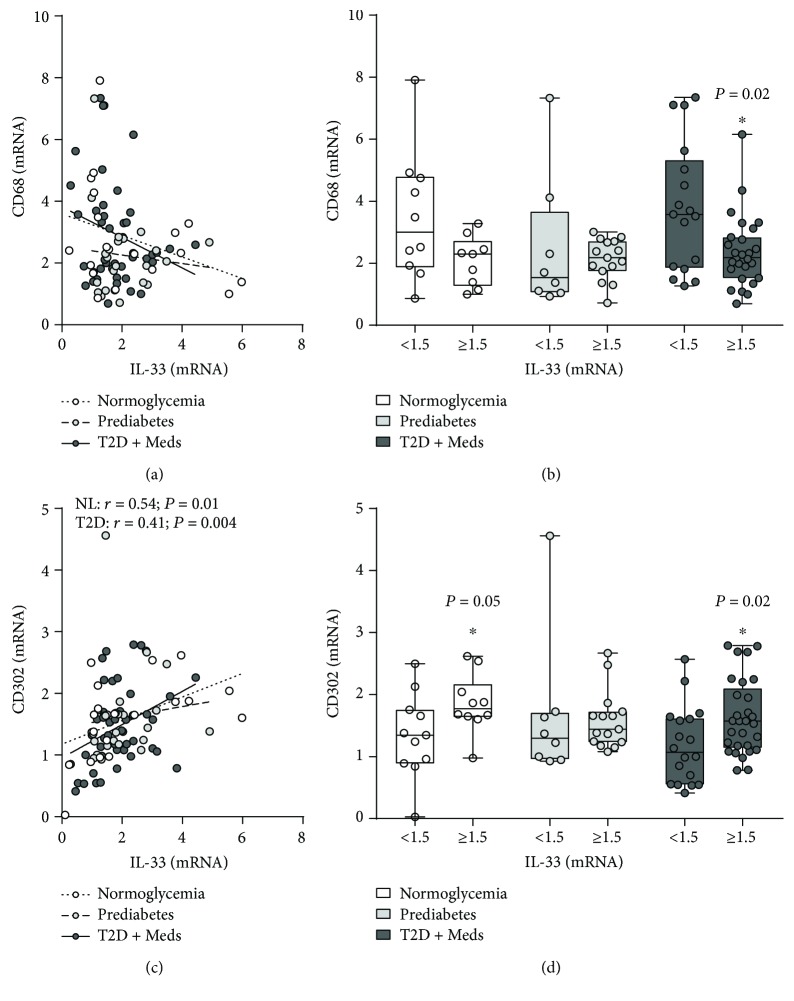
Association between adipose tissue IL-33 and CD68 and CD302 in individuals with varying degrees of glycemia. (a) IL-33 was inversely correlated (although statistically nonsignificant) with CD68 in individuals with normoglycemia (*n* = 19), prediabetes (*n* = 23), and T2D (*n* = 44). (b) Higher levels of IL-33 were associated with significantly lower CD68 in individuals with T2D but not in those with normoglycemia or prediabetes. (c) IL-33 was directly correlated with CD302 in individuals with normoglycemia (*n* = 21) and T2D (*n* = 47) but not in those with prediabetes (*n* = 23). (d) Higher levels of IL-33 were associated with higher CD302 in individuals with normoglycemia (although statistically nonsignificant) and T2D (statistically significant) but not in those with prediabetes. IL-33: interleukin-33; CD68: cluster of differentiation 68; CD302: cluster of differentiation 302; NL: normoglycemia; T2D: type 2 diabetes; Meds: glucose-lowering medications.

**Figure 5:**
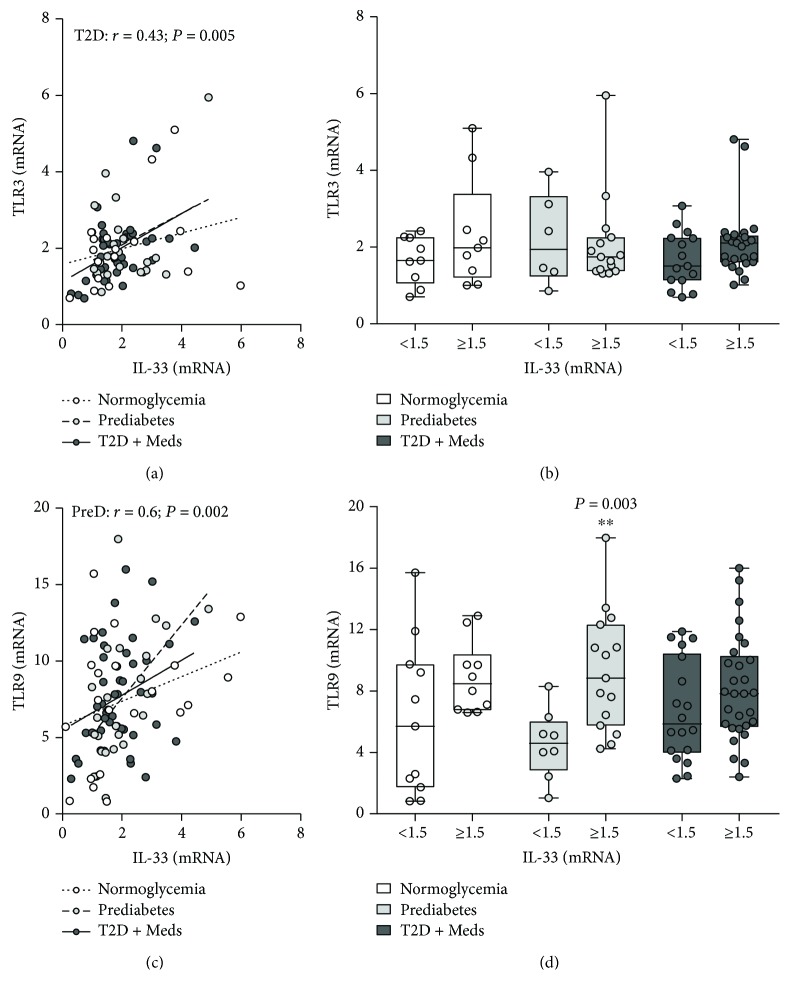
Association between adipose tissue IL-33 and TLR3 and TLR9 in individuals with varying degrees of glycemia. (a) IL-33 was directly correlated with TLR3 in individuals with T2D (*n* = 40) but not in those with normoglycemia (*n* = 18) or prediabetes (*n* = 21). (b) Higher levels of IL-33 were associated with higher TLR3 in individuals with T2D (although statistically nonsignificant) but not in those with normoglycemia or prediabetes. (c) IL-33 was directly correlated with TLR9 in individuals with prediabetes (*n* = 23) but not in those with normoglycemia (*n* = 21) or T2D (*n* = 47). (d) Higher levels of IL-33 were associated with significantly higher TLR9 in individuals with prediabetes but not in those with normoglycemia or T2D. IL-33: interleukin-33; TLR3: toll-like receptor 3; TLR9: toll-like receptor 9; PreD: prediabetes; T2D: type 2 diabetes; Meds: glucose-lowering medications.

The association between adipose tissue IL-33 and macrophage markers, including pattern recognition receptors (PRRs) and chemokine receptors, was assessed in individuals with normoglycemia, prediabetes, and T2D. There was no correlation between IL-33 and CD68 (a universal macrophage marker); however, comparative analysis revealed that higher levels of IL-33 were associated with significantly lower CD68 (*P* = 0.02; median 2.19, *n* = 27 vs. median 3.57, *n* = 17) in individuals with T2D (Figures 4(a) and 4(b)). There was no correlation between IL-33 and CD11c, CD86, CD163, and chemokine receptors CCR1, CCR2, and CCR5 (data not shown). With regard to PRRs, IL-33 was directly correlated with the C-type lectin receptor CD302 in individuals with normoglycemia (*r* = 0.54; *P* = 0.01; *n* = 21) and T2D (*r* = 0.41; *P* = 0.004; *n* = 47) but not in those with prediabetes. Comparative analysis confirmed that higher levels of IL-33 tended to be associated with higher CD302 expression in individuals with normoglycemia (although this did not reach statistical significance, *P* = 0.05; median 1.77, *n* = 10 vs. median 1.3, *n* = 11) and T2D (*P* = 0.02; median 1.57, *n* = 29 vs. median 1.07, *n* = 18) but not in those with prediabetes (Figures 4(c) and 4(d)). There was no association between IL-33 and CLEC7A among individuals with normoglycemia, prediabetes, or T2D (data not shown). There was no correlation between IL-33 and toll-like receptor (TLR) 2, TLR4, TLR7, TLR8, and TLR10 among individuals with normoglycemia, prediabetes, or T2D. However, IL-33 was directly correlated with TLR3 (*r* = 0.43; *P* = 0.005; *n* = 40) in individuals with T2D, although comparative analysis did not reach statistical significance (Figures 5(a) and 5(b)). Interestingly, IL-33 was directly correlated with TLR9 (*r* = 0.6; *P* = 0.002; *n* = 23) in individuals with prediabetes; and comparative analysis confirmed that higher levels of IL-33 were associated with significantly higher TLR9 expression (*P* = 0.003; median 8.84, *n* = 15 vs. median 4.6, *n* = 8) (Figures 5(c) and 5(d)).

### 3.4. Association between Adipose Tissue IL-33 and Mediators of Inflammation and Immune Regulation

**Figure 6:**
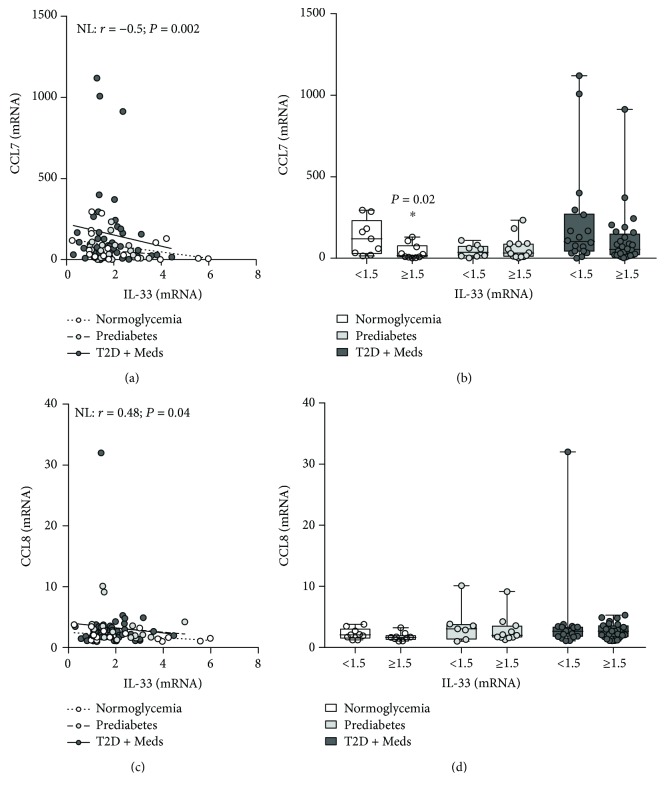
Association between adipose tissue IL-33 and CCL7 and CCL8 in individuals with varying degrees of glycemia. (a) IL-33 was inversely correlated with CCL7 in individuals with normoglycemia (*n* = 19) but not in those with prediabetes (*n* = 22) or T2D (*n* = 46). (b) Higher levels of IL-33 were associated with significantly lower CCL7 in individuals with normoglycemia but not in those with prediabetes or T2D. (c) IL-33 was inversely correlated with CCL8 in individuals with normoglycemia (*n* = 18) but not in those with prediabetes (*n* = 18) or T2D (*n* = 43). (d) Higher levels of IL-33 were associated with lower CCL8 in individuals with normoglycemia (although statistically nonsignificant) but not in those with prediabetes or T2D. IL-33: interleukin-33; CCL7: chemokine (C-C motif) ligand 7; CCL8: chemokine (C-C motif) ligand 8; NL: normoglycemia; T2D: type 2 diabetes; Meds: glucose-lowering medications.

**Figure 7:**
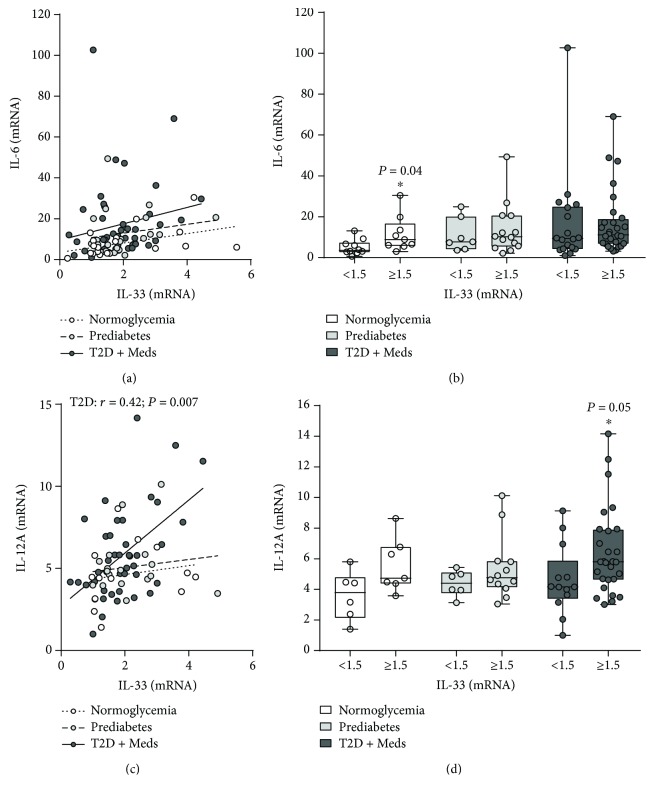
Association between adipose tissue IL-33 and IL-6 and IL-12A in individuals with varying degrees of glycemia. (a) IL-33 was directly correlated with IL-6 (although statistically nonsignificant) in individuals with normoglycemia (*n* = 19), prediabetes (*n* = 21), and T2D (*n* = 46). (b) Higher levels of IL-33 were associated with significantly higher IL-6 in individuals with normoglycemia but not in those with prediabetes or T2D. (c) IL-33 was directly correlated with IL-12A in individuals with T2D (*n* = 41) but not in those with normoglycemia (*n* = 13) or prediabetes (*n* = 18). (d) Higher levels of IL-33 were associated with significantly higher IL-12A in individuals with T2D (although statistically nonsignificant) but not in those with normoglycemia or prediabetes. IL-33: interleukin-33; IL-6: interleukin-6; IL-12A: interleukin-12A; T2D: type 2 diabetes; Meds: glucose-lowering medications.

The association between adipose tissue IL-33 and inflammatory cytokines and chemokines was evaluated in individuals with normoglycemia, prediabetes, and T2D. There was no association between IL-33 and inflammatory cytokines including IL-1*β*, TNF-*α*, IL-8, IL-18, or IL-23A. Similarly, there was no association between IL-33 and chemokine (C-C motif) ligand (CCL) 2 (CCL2), CCL5, CCL11, CCL15, CCL19, CCL20, (C-X-C motif) ligand (CXCL) 8, CXCL9, and CXCL10, among individuals with normoglycemia, prediabetes, or T2D. However, IL-33 was inversely correlated with CCL7 in individuals with normoglycemia (*r* = −0.5; *P* = 0.02; *n* = 19) but not in those with prediabetes or T2D. Comparative analysis confirmed that higher levels of IL-33 were associated with significantly lower CCL7 in individuals with normoglycemia (*P* = 0.02; median 16.7, *n* = 10 vs. median 118.8, *n* = 9) but not in those with prediabetes or T2D (Figures 6(a) and 6(b)). Similarly, IL-33 was inversely correlated with CCL8 in individuals with normoglycemia (*r* = −0.48; *P* = 0.04; *n* = 18) but not in those with prediabetes or T2D. However, although higher levels of IL-33 were associated with lower CCL8 in individuals with normoglycemia, this did not reach statistical significance (Figures 6(c) and 6(d)). Interestingly, although IL-33 was not correlated with IL-6 in individuals with normoglycemia, prediabetes, or T2D, higher levels of IL-33 were associated with significantly higher IL-6 in individuals with normoglycemia (*P* = 0.04; median 8.86, *n* = 9 vs. median 3.5, *n* = 10) but not in those with prediabetes or T2D (Figures 7(a) and 7(b)). Furthermore, IL-33 was directly correlated with IL-12A (IL-12B was not tested) in individuals with T2D (*r* = 0.42; *P* = 0.007; *n* = 41) but not in those with normoglycemia or prediabetes. Among individuals with T2D, those who had higher levels of IL-33 had higher levels (although statistically nonsignificant) of IL-12A compared with those who had lower levels of IL-33 (*P* = 0.05; median 5.79, *n* = 28 vs. median 4.2, *n* = 13) (Figures 7(c) and 7(d)).

**Figure 8:**
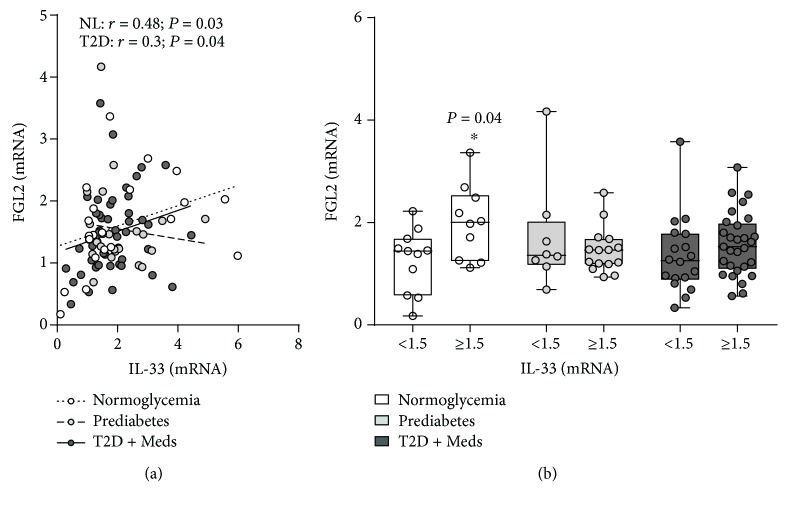
Association between adipose tissue IL-33 and FGL2 in individuals with varying degrees of glycemia. (a) IL-33 was directly correlated with FGL2 in individuals with normoglycemia (*n* = 21) and T2D (*n* = 47) but not in those with prediabetes (*n* = 23). (b) Higher levels of IL-33 were associated with significantly higher FGL2 in individuals with normoglycemia but not in those with prediabetes or T2D. IL-33: interleukin-33; FGL2: fibrinogen-like protein 2; NL: normoglycemia; T2D: type 2 diabetes; Meds: glucose-lowering medications.

Next, we determined whether adipose tissue IL-33 was associated with downstream Th2 cytokines and/or mediators of immune regulation. There was no association between IL-33 and IL-5, IL-10, IL-13, TGF-*β*, FOXP3, or CD127. However, IL-33 was directly correlated with fibrinogen-like protein 2 (FGL2) in individuals with normoglycemia (*r* = 0.48; *P* = 0.03; *n* = 21) and T2D (*r* = 0.3; *P* = 0.04; *n* = 47) but not in those with prediabetes. Among individuals with normoglycemia, those who had higher levels of IL-33 had significantly higher levels of FGL2 (*P* = 0.04; median 2.0, *n* = 10 vs. median 1.44, *n* = 11) compared with those who had lower levels of IL-33 (Figures 8(a) and 8(b)).

### 3.5. Association between Adipose Tissue IL-33 and “Beiging” of Adipose Tissue

**Figure 9:**
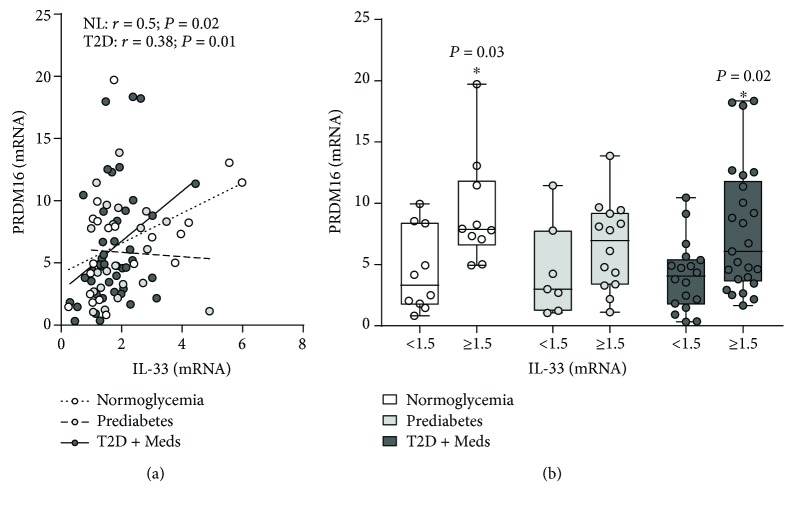
Association between adipose tissue IL-33 and PRDM16 in individuals with varying degrees of glycemia. (a) IL-33 was directly correlated with PRDM16 in individuals with normoglycemia (*n* = 20) and T2D (*n* = 43) but not in those with prediabetes (*n* = 21). (b) Higher levels of IL-33 were associated with significantly higher PRDM16 in individuals with normoglycemia and T2D but not in those with prediabetes. IL-33: interleukin-33; PRDM16: PR domain containing 16; NL: normoglycemia; T2D: type 2 diabetes; Meds: glucose-lowering medications.

We determined whether adipose tissue IL-33 was associated with genes involved in beiging of adipose tissue in individuals with normoglycemia, prediabetes, and T2D. Although IL-33 was not correlated with UCP1 or COX7A1, it was directly correlated with PR domain containing 16 (PRDM16) in individuals with normoglycemia (*r* = 0.5; *P* = 0.02; *n* = 20) and T2D (*r* = 0.38; *P* = 0.01; *n* = 43) but not in those with prediabetes. Similarly, higher levels of IL-33 were associated with significantly higher PRDM16 in individuals with normoglycemia (*P* = 0.03; median 7.9, *n* = 10 vs. median 3.3, *n* = 10) and T2D (*P* = 0.02; median 6.1, *n* = 25 vs. median 4.1, *n* = 18) but not in those with prediabetes (Figures 9(a) and 9(b)).

## 4. Discussion

Adiposity is associated with increased risk of metabolic disease such as insulin resistance and T2D [1, 2]; interestingly, however, some individuals with increased adiposity remain metabolically healthy [3]. It has been demonstrated that the IL-33/ST2 axis plays a protective role against obesity and/or insulin resistance and T2D in animal models [18, 52, 53]. Moreover, there is evidence to suggest that circulating IL-33 may confer protective metabolic effects in humans, more specifically, in individuals with normal weight and glycemia but not with obesity and/or T2D [54]. However, it is not known whether metabolic effects of adipose tissue IL-33 differ among individuals with normoglycemia, prediabetes, and T2D. In the present study, we investigated the expression level of IL-33 (and ST2) in the adipose tissue, as well as its association with various immune and metabolic parameters, in individuals with normoglycemia, prediabetes, and T2D.

We initially asked whether the level of adipose tissue IL-33 (and/or ST2) was differentially expressed among individuals with normoglycemia, prediabetes, or T2D. Our results showed no difference in the level of IL-33 or ST2 among the study groups. Next, we sought to investigate whether adipose tissue IL-33 was differentially associated with overall glycemia (by measuring HbA1c). As was found with circulating IL-33 [54], adipose tissue IL-33 was inversely associated with HbA1c in individuals with normoglycemia. Interestingly, however, an inverse association was also observed in individuals with T2D but not in those with prediabetes; this finding was unexpected since such an association was not observed with circulating IL-33 in individuals with T2D [54]. Given the importance of IL-33/ST2 axis in protection against metabolic disease, we sought to investigate whether IL-33 was differentially associated with ST2 among individuals with normoglycemia, prediabetes, and T2D. Our data showed that IL-33 was directly associated with ST2 in individuals with normoglycemia and T2D but not in those with prediabetes. Taken together, our results suggested that adipose tissue IL-33 was inversely associated with overall glycemia but directly associated with ST2 and, importantly, that these patterns of associations were only observed in individuals with normoglycemia and T2D but not in those with prediabetes. Thus, there appears to be an important dynamic between IL-33 and ST2 in the adipose tissue; one that influences overall glycemia and may be dysfunctional in individuals with prediabetes. Indeed, the ratio of IL-33 to ST2 was inversely associated with HbA1c, although this was only observed in individuals with normoglycemia. Accordingly, we speculate that the dynamic between IL-33 and ST2 may be equipped with a positive feedback loop that promotes protective IL-33/ST2 axis in the adipose tissue. Further, we speculate that the kinetics of innate and/or adaptive immune cell responses, such as Th2 and Treg cells which are protective against metabolic disease, may be regulated by the ratio of IL-33 to ST2. Taking all these observations into consideration, it seems plausible to surmise that the dynamics and biological functions (rather than the level per se) of endogenous adipose tissue IL-33 and ST2 may play a role in overall glycemia in humans (and irrespective of its influence on body weight).

Since prediabetes is an early-stage metabolic disorder which, if left unmanaged, often progresses to T2D, one would have expected to observe a similar dynamic between IL-33/ST2 and HbA1c among individuals with prediabetes and T2D; however, this was not the case. The lack of an association between IL-33 and overall glycemia in individuals with prediabetes may point toward a potential dysfunction in the IL-33/ST2 axis that may occur early during the development of metabolic disorders/T2D and its potential reversal with glucose-lowering medications as may be occurring in individuals with T2D. In this regard, glucose-lowering medications may have played a role in the observed inverse association between adipose tissue IL-33 and HbA1c in individuals with T2D. Thus, the IL-33/ST2 axis may represent a novel target by which glucose-lowering medications confer their metabolic effects in T2D. This notion is supported by the observation that adipose tissue IL-33 was inversely associated with fasting glucose in individuals with T2D and a better glycemic control, which was not observed in individuals with prediabetes despite having a similar HbA1c range. There are various mechanisms by which glucose-lowering medications confer their metabolic effects; in addition to these, we speculate that these medications may also target and restore the dynamics and biological functions of IL-33/ST2 in the adipose tissue of individuals with T2D. Of note, the previous studies have reported that the inhibition of ST2 internalization enhances IL-33-induced cytokine release in epithelial cells [59]. Therefore, one way in which these medications may target/restore the IL-33/ST2 axis in individuals with T2D is by inhibiting ST2 internalization, thereby leading to increased IL-33/ST2 interaction. In addition, it has been demonstrated that IL-33 can be released in either full length or a cleaved form and that processing by caspase-1, caspase-3, or caspase-7 leads to IL-33 inactivation and abrogation of IL-33-mediated responses [60, 61]. Accordingly, it may be possible to speculate that glucose-lowering medications, through inhibition of caspases, may lead to the activation of IL-33 in individuals with T2D.

It is well known that obesity is associated with a state of chronic low-grade inflammation leading to ongoing activation of the innate immune system [62]. The recruitment of immune cells, specifically macrophages, is a key feature in obesity-induced inflammation [63]. Although the phenotype of macrophages in the adipose tissue is highly dependent on the microenvironment [64], macrophages can generally be divided into two main phenotypes: the alternatively activated (M2) macrophages which are metabolically protective and the classically activated (M1) macrophages which are proinflammatory. However, macrophages can have mixed states that do not fall strictly under the M1/M2 phenotypes [65], and thus, phenotyping may not adequately capture the inflammatory state of the adipose tissue. IL-33 has been shown to be metabolically protective in murine models of obesity in which various mechanisms have been described [18, 52, 53]. One mechanism by which IL-33 exerts these protective metabolic effects is through polarization of resident macrophages toward M2 phenotype and by recruitment of immune cells that are involved in type 2 immune responses [18, 52]. Regarding our findings, given that adipose tissue IL-33 was inversely associated with HbA1c, we speculated that this may have been due to IL-33-induced reduction in adipose tissue inflammation. To address this possibility, we attempted to investigate the association between adipose tissue IL-33 and a select number of markers known to be associated with inflammation and immune regulation. Our data showed that there was no association between IL-33 and CD68 (a universal marker of macrophages), CD86 (regarded as an M1 marker [66]), CD11c (upregulated in M1), or CD163 (upregulated in M2 but can also be expressed by M1; the M2 marker CD206 [67] was not tested). Of note, although there was no correlation between IL-33 and CD68, higher levels of IL-33 were associated with lower CD68 in individuals with T2D which may suggest reduced monocyte/macrophage recruitment in the adipose tissue. Importantly, since this association was only observed in individuals with T2D, this may have been potentiated by glucose-lowering medications. It was not possible to ascertain whether IL-33 was associated with an increased M2 phenotype since this would have required a wide range of markers ascribed to M1 and M2 phenotypes. Future studies addressing this possibility, along with the effect of glucose-lowering medications on M1 versus M2, are required.

In addition, we investigated the association between adipose tissue IL-33 and PRRs, including the C-type lectin receptor CD302 and a range of toll-like receptors. In humans, CD302 is expressed by myeloid-derived cells such as monocytes, macrophages, and myeloid dendritic cells (DCs) [68]. We found a direct association between IL-33 and CD302 in individuals with normoglycemia and T2D but not in those with prediabetes. The previous studies have shown that the activation of monocyte-derived macrophages or DCs leads to decreased expression of CD302 [68]. Thus, the observed direct association between IL-33 and CD302 may reflect a reduced state of macrophage activation, which may translate into reduced adipose tissue inflammation. On the other hand, the previous *in vitro* studies on M1/M2 macrophage differentiation have shown that M2 macrophages (differentiated with IL-4) increased the expression of CD302 by almost 2-fold when compared with M0 macrophages. Although M1 macrophages also express CD302, cell surface expression was greater in M2 than M1 [69]. Therefore, the direst association between IL-33 and CD302 may reflect the preferential differentiation of M2 macrophages in the presence of increased IL-33 (although it may reflect increased CD302 on myeloid DCs as well). However, these possibilities need to be explored further with mechanistic as well as clinical studies. Out of all the TLRs tested, IL-33 was directly correlated with TLR3 and TLR9 in individuals with T2D and prediabetes, respectively. TLR3 influences glucose homeostasis and beta-cell insulin secretion [70], whereas TLR9 prevents response to self/host DNA, thereby preventing autoinflammation while facilitating access to pathogen/viral DNA [71, 72]. Therefore, there appears to be some potential dynamic between IL-33 and the TLR system, specifically TLR3 and TLR9, during the development of metabolic disease, which may be impacted by glucose-lowering medications.

Studies in animal models suggest that Th1/Tc1 are detrimentally involved in the attraction and differentiation of adipose tissue macrophages, whereas Th2 cells and, mainly, Treg cells are protective [73–75]. Our data showed no association between IL-33 and the inflammatory mediators TNF-*α*, IL-1*β*, IL-8, IL-18, or IL-23A. Interestingly, however, a direct association was observed between IL-33 and IL-12A in individuals with T2D but not in those with normoglycemia or prediabetes. IL-12A can form the *α*-subunit (IL-12p35) of proinflammatory IL-12 (the *β* subunit being IL-12p40) or immunosuppressive IL-35 (the *β* subunit being Ebi3) [76, 77]; however, since the *β* subunits of the cytokines IL-12 and IL-35 were not assessed, it was not possible to get a clearer idea as to which of these cytokines were associated with IL-33. Nonetheless, considering that IL-33 is metabolically protective [18, 52, 53], combined with our finding that IL-33 was inversely associated with HbA1c in individuals with T2D, we speculate that this association between IL-33 and IL-12A may reflect its association with IL-35 rather than IL-12. However, this is mere speculation, and further studies are required to specifically assess the association between IL-33 and IL-12 versus IL-35.

It has been shown that Th2 and Treg cells play a protective role in the context of adipose tissue inflammation [73–75]. IL-33 induces Th2 cytokine production, mainly IL-5 and IL-13 [36], and has also been associated with Treg cell responses [78–80]. Therefore, we assessed whether IL-33 was associated with Th2 cytokines and/or mediators involved in immune regulation. Our data showed that IL-33 was not associated with Th2 cytokines IL-5 and IL-13 or with mediators involved in immune regulation, including IL-10, TGF-*β*, FOXP3, and CD127. Interestingly, however, IL-33 was directly associated with FGL2 in individuals with normoglycemia and T2D but not in those with prediabetes. Previously, FGL2 had been demonstrated to promote regulatory T cell function, to suppress Th1 and enhance Th2 skewing, and to downregulate antigen presentation [81–85]. Therefore, our finding may point toward a novel mechanism by which IL-33 promotes Th2 and Treg cell responses, possibly through interaction with ST2.

Moreover, the previous studies have shown that white adipose tissue can convert to a brown-like depot termed “beige” adipose [7, 8], which mainly occurs in subcutaneous white adipose tissue depots [7, 8]. Beige adipose tissue dissipates energy and influences glucose and VLDL-TG metabolism [9, 10], potentially protecting against metabolic diseases, such as T2D. Although IL-33 has been implicated in promoting beiging of white adipose tissue in animal models [86, 87], no studies have assessed whether IL-33 is associated with beiging of adipose tissue in humans. Given that beige adipose can influence glucose metabolism [9, 10], we speculated that the observed inverse association between IL-33 and HbA1c may be due to IL-33-mediated beiging of white adipose tissue. To this end, we examined the association between IL-33 and genes involved in beiging of white adipose tissue. Accordingly, IL-33 was directly associated with PRDM16 but not with UCP1 or COX7A1 in individuals with normoglycemia and T2D but not in those with prediabetes. Therefore, our results suggest that IL-33 may play a role in glucose metabolism through a mechanism that may involve PRDM16.

## 5. Conclusions

Endogenous adipose tissue IL-33 and its dynamics with ST2 may play a role in overall glycemia in humans. The lack of an association between IL-33 and overall glycemia in individuals with prediabetes but not in those with T2D may suggest a potential dysfunction in the IL-33/ST2 axis in early-stage metabolic disorders and its potential reversal with glucose-lowering medications, as may be occurring in individuals with T2D. Therefore, our data suggest that it may be beneficial to start glucose-lowering management in the early stages of metabolic disease development. Furthermore, exogenous administration of IL-33 to individuals with prediabetes (and/or T2D) may promote reversal of metabolic derangement. We believe that these results will form a basis for novel mechanistic and clinical studies in people with prediabetes and T2D.

## Figures and Tables

**Table 1:** Anthropometric and biochemical characteristics of the study groups.

Participants	Normoglycemia	Prediabetes	Type 2 diabetes	*P* value^∗^
Total number (*n*)	21	23	47	-
Age (years)	39 ± 11	46 ± 12.1	54 (23, 72)	<0.0001
Body mass index (kg/m^2^)	30.1 ± 5.2	31.16 ± 5.54	31.3 ± 3.79	0.38
Percentage body fat (%)	34.4 ± 6.5 (*n* = 20)	34.92 ± 6.7 (*n* = 18)	35.44 ± 5.59 (*n* = 37)	0.85
Soft lean mass (%)	59.97 ± 6.41 (*n* = 20)	59.45 ± 6.68 (*n* = 18)	58.96 ± 5.58 (*n* = 37)	0.85
Total body water (%)	47.25 ± 4.65 (*n* = 20)	46.88 ± 4.87 (*n* = 18)	46.53 ± 4.05 (*n* = 37)	0.87
SLM-to-PBF ratio	1.64 (1.1, 3.7) (*n* = 20)	1.8 ± 0.6 (*n* = 18)	1.58 (1.07, 3.19) (*n* = 37)	0.85
Waist-to-hip ratio	0.88 (0.48, 1.07) (*n* = 20)	0.91 ± 0.12 (*n* = 19)	0.94 (0.77, 2.34) (*n* = 37)	0.12
Fasting glucose (mmol/L)	5.1 ± 0.38	5.3 ± 0.54	8.2 (4.6, 17.7)	<0.0001
HbA1c (%)^†^	5.3 (4.4, 5.6) (*n* = 20)	5.87 ± 0.14	8.2 ± 1.65 (4.9, 12.3)	<0.0001
Total cholesterol (mmol/L)	4.7 ± 0.8	5.4 ± 0.88	4.63 (2.5, 8.5) (*n* = 46)	0.038
HDL cholesterol (mmol/L)^‡^	1.16 (0.88, 2.24)	1.29 ± 0.3	1.16 ± 0.3 (*n* = 45)	0.18
LDL cholesterol (mmol/L)^§^	2.99 ± 0.66	3.62 ± 0.75	2.8 ± 1.16 (*n* = 44)	0.003
Triglyceride (mmol/L)	0.79 (0.43, 2.93)	1.1 ± 0.44	1.39 (0.36, 7.63) (*n* = 46)	0.0009
hsCRP (*μ*g/mL)^¶^	4.48 ± 3.24 (*n* = 12)	3.85 (0.72, 8.14) (*n* = 16)	4.72 (0.94, 17.92) (*n* = 27)	0.4
White blood cell count (10^9^/L)	5.7 ± 1.35	6.8 ± 2.17 (*n* = 21)	7.4 ± 2.1 (*n* = 45)	0.006

Data shown represent either the standard deviation of the mean or the median (min, max) as indicated. ^∗^Nonparametric Kruskal-Wallis test of the medians. ^†^Glycated hemoglobin. ^‡^High-density lipoprotein. ^§^Low-density lipoprotein. ^¶^High-sensitivity C-reactive protein.

## Data Availability

The raw data of the study can be provided upon request with maintenance of confidentiality, privacy, and anonymity of the research participants.

## Supplementary Materials

Supplementary Materials A table showing (1) the assay IDs of the analyzed genes and (2) the *P* values (Kruskal-Wallis test) depicting any differences in the gene expression of the analyzed mediators among the three groups.
