# Supplementary material for: Association between Adipose Tissue Interleukin-33 and Immunometabolic Markers in Individuals with Varying Degrees of Glycemia
Source: Dis Markers. 2019 Apr 3;2019:7901062. doi: 10.1155/2019/7901062 (PMC6470453; doi:10.1155/2019/7901062)
Supplement: Supplementary Materials — A table showing (1) the assay IDs of the analyzed genes and (2) the P values (Kruskal-Wallis test) depicting any differences in the gene expression of the analyzed mediators among the three groups. [file 7901062.f1.pdf]

Gene	Assay ID	Gene expression differences among individuals with varying degrees of glycemia (**P*-value, Kruskal-Wallis test)
ST2	Hs00545033_m1	$P \geq 0.05$
IL-5	Hs01548712_g1	$P \geq 0.05$
**IL-6**	**Hs00985639_m1**	***P* = 0.047**
IL-8	Hs00174103_m1	$P \geq 0.05$
IL-10	Hs00961622_m1	$P \geq 0.05$
IL-12A	Hs01073447_m1	$P \geq 0.05$
**IL-13**	**Hs00174379_m1**	***P* = 0.02**
IL-18	Hs01038788_m1	$P \geq 0.05$
IL-23A	Hs00900828_g1	$P \geq 0.05$
IL-33	Hs00369211_m1	$P \geq 0.05$
IL-1β	Hs01555410_m1	$P \geq 0.05$
TNF-α	Hs01113624_g1	$P \geq 0.05$
TGF-β	Hs00820148_g1	$P \geq 0.05$
FGL2	Hs00173847_m1	$P \geq 0.05$
PRDM16	Hs00922674_m1	$P \geq 0.05$
UCP1	Hs00222453_m1	$P \geq 0.05$
COX7A1	Hs03045102_g1	$P \geq 0.05$
FOXP3	Hs01085834_m1	$P \geq 0.05$
CD11c	Hs00174217_m1	$P \geq 0.05$
CD68	Hs02836816_g1	$P \geq 0.05$
CD86	Hs01567026_m1	$P \geq 0.05$
CD127	Hs00902334_m1	$P \geq 0.05$
CD163	Hs00174705_m1	$P \geq 0.05$

CD302	Hs00994886_m1	$P \geq 0.05$
CLEC7A	Hs01902549_s1	$P \geq 0.05$
TLR2	Hs01872448_s1	$P \geq 0.05$
TLR3	Hs01551078_m1	$P \geq 0.05$
TLR4	Hs00152939_m1	$P \geq 0.05$
TLR7	Hs01933259_s1	$P \geq 0.05$
TLR8	Hs00152972_m1	$P \geq 0.05$
TLR9	Hs00370913_s1	$P \geq 0.05$
TLR10	Hs01935337_s1	$P \geq 0.05$
**CCL2**	**Hs00234140_m1**	$\boldsymbol{P = 0.03}$
CCL5	Hs00982282_m1	$P \geq 0.05$
CCL7	Hs00171147_m1	$P \geq 0.05$
CCL8	Hs04187715_m1	$P \geq 0.05$
CCL11	Hs00237013_m1	$P \geq 0.05$
CCL15	Hs00361122_m1	$P \geq 0.05$
**CCL19**	**Hs00171149_m1**	$\boldsymbol{P = 0.002}$
CCL20	Hs01011368_m1	$P \geq 0.05$
CXCL9	Hs00171065_m1	$P \geq 0.05$
**CXCL10**	**Hs01124251_g1**	$\boldsymbol{P = 0.025}$
CCR1	Hs00928897_s1	$P \geq 0.05$
**CCR2**	**Hs00704702_s1**	$\boldsymbol{P = 0.03}$
**CCR5**	**Hs99999149_s1**	$\boldsymbol{P = 0.016}$
